# Urinary incontinence in women: assessment with the aid of standardized nursing terminologies NANDA-I and NOC

**DOI:** 10.1590/0034-7167-2022-0714

**Published:** 2023-11-27

**Authors:** Liana Priscilla Lima de Melo, Lívia Maia Pascoal, Isaura Letícia Tavares Palmeira Rolim, Francisca Aline Arrais Sampaio Santos, Floriacy Stabnow Santos, Marcelino Santos, Francisca Elisângela Teixeira Lima, Alana Gomes de Araújo Almeida

**Affiliations:** I Universidade Federal do Maranhão. São Luís, Maranhão, Brazil; II Universidade Federal do Maranhão. Imperatriz, Maranhão, Brazil; III Universidade Federal do Ceará. Fortaleza, Ceará, Brazil

**Keywords:** Urinary Incontinence, Standardized Nursing Terminology, Nursing Diagnosis, Outcome Assessment, Health Care, Women, Incontinência Urinaria, Terminología Normalizada de Enfermería, Diagnóstico de Enfermería, Evaluación de Resultado en la Atención de Salud, Mujeres, Incontinência Urinária, Terminologia Padronizada em Enfermagem, Diagnóstico de Enfermagem, Avaliação de Resultados em Cuidados de Saúde, Mulheres.

## Abstract

**Objectives:**

to assess urinary impairment in incontinent women with the aid of standardized nursing terminologies NANDA-I and NOC.

**Methods:**

a cross-sectional study, carried out with 97 women attending the gynecology outpatient clinic of a university hospital. Data collection took place using a form that contained information about NANDA-I diagnoses related to urinary incontinence and NOC Urinary Continence indicators. Statistical analysis was performed to assess the impairment of NOC indicators in the presence of NANDA-I nursing diagnoses.

**Results:**

diagnosis Mixed Urinary Incontinence was the most prevalent (43.3%), and, in its presence, the most compromised indicators were voids in appropriate receptacle, gets to toilet between urge and passage of urine and empties bladder completely.

**Conclusions:**

urinary impairment was worse in women with elements of stress and urge urinary incontinence.

## RESUMO

**Objetivos:**

avaliar o comprometimento urinário em mulheres incontinentes com o auxílio das terminologias padronizadas em enfermagem NANDA-I e NOC.

**Métodos:**

estudo transversal, realizado com 97 mulheres em atendimento no ambulatório de ginecologia de um hospital universitário. A coleta de dados ocorreu por meio de formulário que continha informações sobre diagnósticos da NANDA-I relacionados à incontinência urinária e indicadores do resultado NOC Continência Urinária. Foi realizada análise estatística para avaliar o comprometimento dos indicadores da NOC na presença dos diagnósticos de enfermagem da NANDA-I.

**Resultados:**

o diagnóstico Incontinência Urinária Mista foi o mais prevalente (43,3%), e, na sua presença, os indicadores mais comprometidos foram urina em recipiente apropriado, chega ao banheiro entre o desejo e a passagem de urina e esvazia a bexiga completamente.

**Conclusões:**

o comprometimento urinário demonstrou-se pior nas mulheres com elementos de incontinência urinária de esforço e urgência.

## RESUMEN

**Objetivos:**

evaluar la afectación urinaria en mujeres incontinentes con la ayuda de las terminologías estandarizadas de enfermería NANDA-I y NOC.

**Métodos:**

estudio transversal, realizado con 97 mujeres que asisten a la consulta externa de ginecología de un hospital universitario. La recolección de datos se realizó a través de un formulario que contenía información sobre los diagnósticos de la NANDA-I relacionados con la incontinencia urinaria y los indicadores del resultado NOC Continencia Urinaria. Se realizó análisis estadístico para evaluar el deterioro de los indicadores NOC en presencia de diagnósticos de enfermería NANDA-I.

**Resultados:**

el diagnóstico Incontinencia Urinaria Mixta fue el más prevalente (43,3%), y, en su presencia, los indicadores más comprometidos fueron orina en recipiente adecuado, llegar al baño entre el deseo y el paso de la orina y vaciar completamente la vejiga.

**Conclusiones:**

la afectación urinaria fue peor en mujeres con elementos de estrés e incontinencia urinaria de urgencia.

## INTRODUCTION

Urinary incontinence (UI) is a serious social and health problem, defined as any involuntary loss of urine that can be classified according to NANDA-International, Inc. (NANDA-I) in stress UI, urge UI, mixed UI and disability-associated UI^(1)^. This voiding disorder can affect individuals of all ages, both sexes and from all social and economic levels^(2)^. However, it is more frequent in women than in men^(3)^, affecting approximately 10 to 25% of women over 30 years of age, with intervals ranging from 30% to 50% at 50 years of age^(4)^.

The risk factors for the development of UI in women are multiple and deserve special attention, such as age at menopause, presence of comorbidities such as diabetes mellitus (DM) and hypertension, natural births in quantitative terms and multiplicity of fetuses^(5)^. Thus, early diagnosis of UI is paramount, consisting of patient history, physical examination and other complementary tests, such as urine and post-void residue, to exclude other conditions that require specialized care^(6)^.

Regarding treatment, conservative and surgical are the types recommended for UI, the latter being indicated, mainly, when conservative treatment fails and for the most complicated cases of UI^(7)^. Conservative approaches to UI treatment include behavioral therapy and pelvic floor training, which should be used as the first option, and have low rates of side effects^(8)^.

In this sense, the treatment of people with UI involves a multidisciplinary team, and nurses, through the Nursing Process (NP), a methodological instrument that guides professional care, have the function of obtaining information on the health-disease process, interpreting the data collected, determining the results to be achieved, carrying out nursing interventions and assessing responses after interventions, whether in the home or hospital context^(9-10)^.

As facilitating tools for NP implementation, standardized terminologies in nursing are considered essential, as they provide quality to nursing records^(11)^. Therefore, the use of nursing taxonomies in clinical practice, such as NANDA-I, the Classification of Nursing Interventions (NIC) and the Classification of Nursing Outcomes (NOC), is considered a priority target for the scientific community, as it allows a better understanding of aspects of patients through terms that can be understood and shared by nurses around the world, in addition to highlighting the focus of nursing care^(12)^.

The NOC, in particular, arose from the need to implement a specific language that would allow assessing the NP^(13)^. Thus, it is understood that this taxonomy enables a standardized and, at the same time, individualized assessment of patients, since it separately assesses the degree of commitment of each indicator analyzed, thus allowing the planning of care according to each characteristic presented^(13)^.

Despite its importance, studies that address the theme of UI associated with the use of standardized terminologies in nursing are less frequent, especially the NOC taxonomy, when compared to those of NANDA-I^(14)^.

As UI causes significant changes in women’s lives, in the physical and psychosocial dimensions, and studies that use NOC Urinary Continence indicators to assess this population group are scarce^(15)^, the existing gap in the literature is evidenced, pointing to the need for studies in the area, in order to determine criteria and improvements in urinary assessment practice, with the use of nursing outcomes in incontinent women. Given this context, the following questions arose: which NOC indicators indicate greater urinary impairment in women with involuntary loss of urine? What is the most frequent nursing diagnosis related to UI in these women?

## OBJECTIVES

To assess urinary impairment in incontinent women with the aid of standardized nursing terminologies NANDA-I and NOC.

## METHODS

### Ethical aspects

The study was conducted in accordance with national and international ethics guidelines, and approved by the Research Ethics Committee of the *Universidade Federal do Maranhão* (UFMA), whose opinion is attached to this submission. The participants of this research gave consent by signing the Informed Consent Form.

### Study design, place and period

This is an analytical cross-sectional study, developed according to the STrengthening the Reporting of OBservational studies in Epidemiology (STROBE) recommendations with women who had UI. The survey was carried out between October 2021 and May 2022 at the gynecology outpatient clinic of a university hospital in northeastern Brazil.

### Population, sample, and inclusion and exclusion criteria

The population of this research consisted of women treated at the outpatient clinic of a university hospital. Women aged over 18 years who had symptoms related to involuntary loss of urine were included. Those who were pregnant, who had communication barriers, degenerative neurological diseases or who had undergone a surgical procedure for treatment of UI were excluded.

To define the sample size, the calculation for a finite population with known proportions was used, based on a 95% confidence interval (α = 0.05), 50% (p=0.50) estimated prevalence and 5% sampling error, resulting in a minimum sample of 92 participants. However, in this research, the sample consisted of 97 women. The non-probabilistic technique of sequential sampling was used to capture the women in this study^(16)^.

### Study protocol

Data collection was performed through interviews, using a form consisting of three parts: part 1 - sociodemographic variables, general health data, gynecological-obstetric history and eating habits; part 2 - classification of women according to NANDA-I taxonomy II regarding the presence of nursing diagnoses Stress Urinary Incontinence (SUI), Urge Urinary Incontinence (UUI), or Mixed Urinary Incontinence (MUI)^(1)^, considering that nursing diagnoses’ interference was determined by the researcher herself at the time of data collection; part 3 - NOC Urinary Continence indicators, to assess urinary impairment in women who had involuntary loss of urine^(17)^.

In this study, the NOC Urinary Continence was chosen, due to the relationship that it has with the most common nursing diagnoses related to UI, verified by consulting the book “NANDA, NOC, and NIC Linkages”^(18)^, in addition to the scarcity of studies that assess the impairment of involuntary loss of urine through nursing taxonomies.

The conceptual and operational definitions of the 19 indicators of this outcome were prepared by the researcher herself, through searches in the scientific literature on the subject, then submitted to the validity process with 26 specialists in the areas of standardized terminologies in nursing and/or stomatherapy (UI). Only after the validity process by experts, the indicators of this outcome were used for clinical applicability.

In this research, the indicator post void residual > 100-200 milliliters was not used with the participants, due to the impossibility of assessment through interviews. The first thirteen indicators contained in the NOC were measured using a 5-point Likert scale: never demonstrated (1); rarely demonstrated (2); sometimes demonstrated (3); often demonstrated (4); and consistently demonstrated (5). And the other six indicators, through the 5-point Likert scale, in: consistently demonstrated (1); often demonstrated (2); sometimes demonstrated (3); rarely demonstrated (4); and never demonstrated (5)^(17)^. The identification of urinary impairment in patients was performed by the mean score of each NOC Urinary Continence indicator obtained through the Likert scale.

### Analysis of results, and statistics

Data were analyzed using Statistical Package for the Social Sciences (SPSS) version 24.0. Absolute frequencies, percentages, measures of central tendency and dispersion were determined for the descriptive analysis of variables.

The Mann-Whitney test was used for the analysis of quantitative variables that did not follow a trend towards normality. The adopted significance level was 5% (p < 0.05), with a 95% confidence interval.

## RESULTS

In this research, 97 patients with UI who had a mean age of 50.77 years (SD: 10.21) and a level of education equivalent to high school (56.7%) were assessed. In the overall health data assessment, it was observed that SAH (45.4%) and DM (19.6%) were the most frequent comorbidities. Consequently, the medications used to treat them also predominated, such as antihypertensives (43.3%) and antidiabetics (19.6%), which interfere in the physiology of urination. Regarding eating habits, most reported consuming caffeine (88.7%).

The results obtained in the survey of gynecological-obstetric variables indicated a mean of 2.99 pregnancies per woman (SD:1.61), a mean of 1.96 vaginal births (SD: 1.88) and a mean of 0.74 cesarean births (SD: 0.96). The most frequent type of birth was vaginal (67%), and in 29.9% of cases there was vaginal laceration. Most of assessed women (57.7%) claimed to be in the climacteric/menopause phase.

In the assessment of nursing diagnoses, it was found that MUI was the most frequent (43.3%), followed by SUI (37.1%) and UUI (19.6%).

Table 1 presents the commitment of women with urinary incontinence according to NOC Urinary Continence indicators. The indicators that presented the worst results, i.e., a score between 1 and 2, were: identifies medication that interferes with urinary control (mean = 1.21); urine leakage with sneezing, laughing, or lifting (mean = 2.23); and empties bladder completely (mean = 2.07). In turn, the indicators with the highest means and which the scores were between 4 and 5 were: toilets independently (mean = 4.99); manages clothes independently (mean = 4.96); maintains barrier-free environment for independent toileting (mean = 4.85); wets clothing or bedding during night (mean = 4.67); voids in appropriate receptacle (mean = 4.52); urinary tract infection (mean = 4.38); and recognizes urge to void (mean = 4.23).

**Table 1:** Characterization of urinary impairment in women through nursing outcome Urinary Continence indicators, São Luís, Maranhão, Brazil, 2022

NOC indicators	NOC^*^ Likert scale - Urinary Continence										
	Never demonstrated		Rarely demonstrated		Sometimes demonstrated		Frequently demonstrated		Consistently demonstrated		Mean
	n	%	n	%	n	%	n	%	n	%	
Recognizes urge to void	0	0	7	7.2	13	13.4	28	28.9	49	50.5	4.23
Maintains predictable pattern of voiding	9	9.3	7	7.2	28	28.9	39	40.2	14	14.2	3.43
Responds to urge in timely manner	1	1.0	3	3.1	13	13.4	51	52.6	29	29.9	4.07
Voids in appropriate receptacle	0	0	3	3.1	4	4.1	30	30.9	60	61.9	4.52
Gets to toilet between urge and passage of urine	2	2.1	6	6.2	13	13.4	39	40.2	37	38.1	4.06
Maintains barrier-free environment for independent toileting	3	3.1	1	1.0	0	0	0	0	93	95.9	4.85
Voids greater than 150 millimeters each time	7	7.2	18	18.6	27	27.8	26	26.8	19	19.6	3.33
Starts and stops stream	10	10.3	17	17.5	45	46.4	14	14.4	11	11.3	2.99
Empties bladder completely	37	38.1	32	33.0	19	19.6	2	2.1	7	7.2	2.07
Drinks adequate amount of fluid	7	7.2	22	22.7	22	22.7	21	21.6	25	25.8	3.36
Manages clothes independently	0	0	1	1.0	0	0	1	1.0	95	97.9	4.96
Toilets independently	0	0	0	0	0	0	1	1.0	96	99.0	4.99
Identifies medication that interferes with urinary control	87	89.7	6	6.2	0	0	2	2.1	2	2.1	1.21
	**Consistently demonstrated**		**Frequently demonstrated**		**Sometimes demonstrated**		**Rarely demonstrated**		**Never demonstrated**		**Mean**
Urine leakage between voidings	20	20.6	42	43.3	21	21.6	12	12.4	2	2.1	2.32
Urine leakage with sneezing, laughing, or lifting	41	42.3	19	19.6	17	17.5	14	14.4	6	6.2	2.23
Wets clothing or bedding during night	0	0	1	1.0	6	6.2	17	17.5	73	75.3	4.67
Urinary tract infection	1	1.0	6	6.2	8	8.2	22	22.7	60	61.9	4.38
Wets clothing during day	2	2.1	12	12.4	17	17.5	26	26.8	40	41.2	3.93

* *NOC- Nursing Outcomes Classification.*

Table 2 shows data on the relationship between nursing outcome Urinary Continence and nursing diagnosis Mixed Urinary Incontinence indicators. The results show that women with this nursing diagnosis had the worst NOC scores for gets to toilet between urge and passage of urine (p = 0.022), voids in appropriate receptacle (p = 0.025) and empties bladder completely (p = 0.037).

**Table 2:** Relationship between the scores of nursing outcome Urinary Continence and nursing diagnosis Mixed Urinary Incontinence indicators, São Luís, Maranhão, Brazil, 2022

NOC indicators^*^	Yes (N= 42)		No (N= 55)		*p* value^***^
	Mean	SD^**^	Mean	SD	
Recognizes urge to void	4.19	0.994	4.25	0.907	0.843
Maintains predictable pattern of voiding	3.21	1.335	3.60	0.894	0.220
Responds to urge in timely manner	3.95	0.854	4.16	0.764	0.205
Voids in appropriate receptacle	4.31	0.869	4.67	0.546	0.025
Gets to toilet between urge and passage of urine	3.79	1.094	4.27	0.827	0.022
Maintains barrier-free environment for independent toileting	4.81	0.862	4.87	1.183	0.767
Voids greater than 150 millimeters each time	3.33	1.183	3.33	1.218	0.994
Starts and stops stream	2.83	1.010	3.11	1.149	0.287
Empties bladder completely	1.79	0.951	2.29	1.242	0.037
Drinks adequate amount of fluid	3.14	1.299	3.53	1.260	0.147
Manages clothes independently	4.98	0.154	4.95	0.405	0.859
Toilets independently	4.98	0.154	-	-	0.252
Identifies medication that interferes with urinary control	1.12	0.504	1.27	0.870	0.359
Urine leakage between voidings	2.17	0.824	2.00	1.118	0.357
Urine leakage with sneezing, laughing, or lifting	1.90	1.008	2.47	1.451	0.080
Wets clothing during day	4.00	0.911	3.87	1.277	0.957
Wets clothing or bedding during night	4.79	0.470	4.58	0.738	0.201
Urinary tract infection	4.52	0.804	4.27	1.004	0.278

* NOC - Nursing Outcomes Classification;

** SD - standard deviation;

*** Mann-Whitney test.

Table 3 presents the relationship between nursing outcome Urinary Continence and nursing diagnosis Stress Urinary Incontinence indicators. These data indicate that, in the presence of this diagnosis, patients had a worse score on the indicator urine leakage with sneezing, laughing, or lifting (p= 0.004). In turn, in the absence of this nursing diagnosis, worse scores were found for maintains predictable pattern of voiding (p=0.004), empties bladder completely (p=0.009), gets to toilet between urge and passage of urine (p= 0.013) and responds to urge in timely manner (p= 0.035).

**Table 3:** Relationship between the scores of nursing outcome Urinary Continence and nursing diagnosis Stress Urinary Incontinence indicators, São Luís, Maranhão, Brazil, 2022

NOC indicators^*^	Yes (N= 36)		No (N= 61)		*p* value^***^
	Mean	SD^**^	Mean	SD	
Recognizes urge to void	4.42	0.806	4.11	1.002	0.157
Maintains predictable pattern of voiding	3.89	0.667	3.16	1.241	0.004
Responds to urge in timely manner	4.31	0.668	3.93	0.854	0.035
Voids in appropriate receptacle	4.58	0.604	4.48	0.788	0.682
Gets to toilet between urge and passage of urine	4.36	0.833	3.89	1.018	0.013
Maintains barrier-free environment for independent toileting	4.89	0.667	4.82	0.806	0.618
Voids greater than 150 millimeters each time	3.36	1.175	3.31	1.218	0.875
Starts and stops stream	3.22	1.017	2.85	1.123	0.165
Empties bladder completely	2.44	1.229	1.85	1.046	0.009
Drinks adequate amount of fluid	3.53	1.158	3.26	1.353	0.338
Manages clothes independently	-	-	4.93	0.403	0.275
Toilets independently	-	-	4.98	0.128	0.442
Identifies medication that interferes with urinary control	1.28	0.849	1.16	0.663	0.376
Urine leakage between voidings	2.50	0.971	2.21	1.018	0.125
Urine leakage with sneezing, laughing, or lifting	1.72	1.003	2.52	1.374	0.004
Wets clothing during day	4.00	1.242	3.82	1.066	0.375
Wets clothing or bedding during night	4.75	0.439	4.62	0.734	0.789
Urinary tract infection	4.33	1.042	4.41	0.901	0.846

* NOC - Nursing Outcomes Classification;

** SD - standard deviation;

*** Mann-Whitney test.

Table 4 shows the data corresponding to nursing outcome Urinary Continence and nursing diagnosis Urgent Urinary Incontinence indicators. Based on the results obtained, it was observed that women who did not have the aforementioned diagnosis had the worst NOC scores for the following indicators: urine leakage with sneezing, laughing, or lifting (p=0.001); and voids in appropriate receptacle (p=0.022).

**Table 4:** Relationship between the scores of nursing outcome Urinary Continence and nursing diagnosis Urgent Urinary Incontinence indicators, São Luís, Maranhão, Brazil, 2022

NOC indicators^*^	Yes N= 19		No N= 78		*p* value^***^
	Mean	SD^**^	Mean	SD	
Recognizes urge to void	3.95	1.026	4.11	1.002	0.140
Maintains predictable pattern of voiding	3.05	1.026	3.53	1.125	0.052
Responds to urge in timely manner	3.89	0.875	4.12	0.789	0.327
Voids in appropriate receptacle	4.89	0.375	4.44	0.766	0.022
Gets to toilet between urge and passage of urine	4.11	0.809	4.05	1.018	0.877
Maintains barrier-free environment for independent toileting	4.84	0.688	4.85	0.774	0.812
Voids greater than 150 millimeters each time	3.26	1.327	3.35	1.171	0.841
Starts and stops stream	2.89	1.370	3.01	1.026	0.718
Empties bladder completely	2.00	1.247	2.09	1.130	0.575
Drinks adequate amount of fluid	3.53	1.467	3.32	1.243	0.520
Manages clothes independently	4.84	0.688	4.99	0.113	0.268
Toilets independently	-	-	4.99	0.113	0.622
Identifies medication that interferes with urinary control	1.26	0.933	1.19	0.685	0.945
Urine leakage between voidings	2.32	1.376	2.32	0.904	0.475
Urine leakage with sneezing, laughing, or lifting	3.89	1.049	1.82	1.003	0.001
Wets clothing during day	3.63	1.342	4.00	1.069	0.311
Wets clothing or bedding during night	4.26	1.046	4.77	0.454	0.054
Urinary tract infection	4.16	1.068	4.44	0.920	0.264

* NOC - Nursing Outcomes Classification;

** SD - standard deviation;

*** Mann-Whitney test.

## DISCUSSION

UI is a multifactorial condition in women whose management is considered complex due to the risk factors involved, comorbidities and physiological changes resulting from female aging^(19)^. In this regard, the mean age of the women who participated in the present investigation corroborates a study that sought to verify factors associated with UI by type and severity, whose mean age of women was 56.2 years^(20)^. This result was consistent with the age range indicated by the literature as the last period of the menstrual cycle, between 45 and 55 years, in which the reduction in estrogen levels causes important gynecourological complaints, such as UI^(21)^.

When assessing the comorbidities, it was observed that the results obtained were similar to a study carried out with women in a urogynecology outpatient clinic of a hospital in northeastern Brazil, in which the frequencies of hypertension (61.9%) and DM (28.6%) were more prevalent^(22)^. The existing relationship between these comorbidities and UI can be explained by the continuous use of diuretic drugs to treat hypertension^(23)^ and the reduction in bladder vascularization and hypotrophy of the tissue components of the pelvic floor muscle as a consequence of diabetic cystopathy^(24)^.

As for eating habits, the high consumption of caffeine in incontinent women found in this study corroborated with a research whose consumption was also high (82.7%)^(25)^. This reinforces what the literature points out as a risk factor for UI, due to the fact that this substance causes vesical hyperactivity, mainly in the smooth muscle of the detrusor, increasing involuntary contractions, thus contributing to the involuntary loss of urine^(26)^.

When investigating the obstetric history, it was found that the predominance of vaginal birth was similar to another study in which 56.7% of women after vaginal birth had UI^(27)^. Thus, urinary continence impairment is caused by damage to the pelvic innervation, which overloads the pelvic floor and alters the function of the urethral sphincter, implying urinary losses, mainly due to effort^(28)^.

With regard to vaginal lacerations, the result obtained in this research differed from a study whose prevalence in incontinent women was higher, corresponding to 56.9%(29). This divergence of results can be explained due to multiple factors related to perineal lacerations, such as the duration of the second stage of labor (expulsive period)^(30)^ and other aspects that could not be confirmed by the assessment method used in this study.

Another aspect observed in this investigation was that the percentage of climacteric/menopausal women who had UI was also expressive, which corroborates a study that assessed women with pelvic dysfunctions in which 62.9% of women were in menopause and 88% had UI^(31)^. This relationship can be explained due to the natural reduction in hormone levels in climacteric/menopause, which potentiates pelvic floor disorders, thus contributing to the development of UI^(32)^.

By observing the mean number of pregnancies in the assessed incontinent women, the data obtained converged with a survey carried out with women with MUI with a mean age of 63 years, in which 98.5% were multiparous^(33)^, justifying the reason why the literature points out the emergence of UI during pregnancy and the increased prevalence related to multiple births^(34)^.

Regarding the NANDA-I UI nursing diagnoses, the predominance of nursing diagnosis MUI in this research corroborates another study carried out in Brazil with women over 60 years of age attended at a geriatrics outpatient clinic where MUI was present 63.7% of participants^(35)^. However, this result differed from a clinical research and a comprehensive systematic review whose results indicated SUI as the most frequent^(36)^, with values of 12.6%, followed by MUI with 9.1% and UUI with 5.3%^(37)^. It is believed that this divergence in prevalence values can be explained, mainly, by the methodological approaches used in the studies^(2)^.

Regarding nursing outcome Urinary Continence, the three indicators that were found to be most compromised were identies medication that interferes with urinary control, loss of urine with increased abdominal pressure, and empties bladder completely. With regard to the indicator identifies medication that interferes with urinary control, no similar result was found in the literature for comparison. However, it is noteworthy that the assessed women demonstrated incipient knowledge about UI and its related factors, unaware of medications that may interfere with urinary physiology, which reinforces UI as a stigmatizing condition, making women understand this alteration as something normal with aging^(2,38)^.

The outcome obtained for the indicator urine loss with increased abdominal pressure converges with a study carried out only with women diagnosed with MUI through urodynamic assessment, in which the stress symptom was predominant (57%) to the detriment of the urgency symptoms (43%)^(33)^. This result can also be justified by the impaired relationship between urinary continence and the urethrovesical junction, thus changes that increase intra-abdominal pressure cause an increase in pressure only intravesically and not to the urethra, causing urinary losses^(39)^. As for the indicator empties bladder completely, a similar result was found in women assessed with MUI who had pelvic organ prolapse (41.4%), since the descent of the anterior and/or posterior vaginal wall causes difficulty in completely emptying the bladder^(33)^.

When assessing the relationship between nursing outcome Urinary Continence and nursing diagnosis MUI indicators, it was found that the presence of this diagnosis was related to worse scores of indicators voids in appropriate receptacle, gets to toilet between urge and passage of urine and empties bladder completely. These results corroborate a survey carried out with patients with stroke and MUI, in which the inability to get to toilet in timely manner to avoid urine loss (93.7%), loss of urine before getting to toilet was more prevalent (80.7%); however, showed divergence, as most patients had preserved capacity in complete emptying of the bladder (74.6%)^(40)^. MUI is considered the most serious of the UI due to the difficulty of obtaining positive responses to treatment, as it encompasses conditions of urine loss in various circumstances, being considered complex to manage^(41)^.

With regard to the NOC indicators and nursing diagnosis SUI, it was observed that the presence of this diagnosis was related to worse scores of the indicator loss of urine with increased abdominal pressure. This result was similar to that demonstrated by a study with patients with stroke and UI, in which the involuntary loss of urine in situations of increased abdominal pressure, such as sneezing, coughing and laughing, corresponded, respectively, to 64.4%, 61% and 47.4% in participants with SUI^(40)^. This finding can be explained because, in SUI, there is no mechanism to compensate for the increase in intra-abdominal pressure, and the role of bladder pressure exceeding urethral pressure is responsible for episodes of involuntary loss of urine^(42)^.

As for the NOC indicators and the nursing diagnosis of UUI, it was found that the indicators with the greatest impairment (urine in an appropriate container and loss of urine with increased abdominal pressure) were present in women who did not have this diagnosis. This finding corroborates a content analysis study of nursing diagnoses related to urinary incontinence of NANDA-I, in which these indicators are not related to the defining characteristics and related factors established for UUI^(43)^. Moreover, the results obtained can be justified by the fact that the present research was carried out only with women with preserved cognitive and low prevalence of neurological disorders, which are generally more associated with cases of UUI^(2)^.

### Study limitations

As a limitation, the scarcity of studies with nursing outcome Urinary Continence as a criterion for assessing urinary impairment stands out, preventing the comparison of variables related to NANDA-I nursing diagnoses and NOC indicators. The possibility of diagnostic inference bias should also be considered, considering that the process of inference of nursing diagnoses was performed by the researcher, despite her having experience with nursing taxonomies and stomatherapy (UI). Another limitation presented in this research was the impossibility of assessing the indicator post void residual > 100-200 milliliters with patients, because of the necessary measurement method, which could not be through an interview, but through a physical examination, impossible due to aspects of the COVID-19 pandemic, causing the impossibility of assessing some variables such as laceration and prolapse of pelvic organs. Therefore, some information collected was subjectively assessed through participants’ reports.

### Contributions to health and nursing

This research evidenced the importance of using nursing outcome for assessing women with UI, thus suggesting the need to carry out studies with other target audiences associating this theme with standardized nursing terminologies. Furthermore, the results obtained in this study can help nurses in clinical practice, encouraging them to use the NOC as an assessment tool, thus allowing an adequate elaboration of care plan and contributing to the implementation of assertive nursing interventions.

## CONCLUSIONS

The nursing diagnosis of MUI was the most frequent in the assessed women and the NOC indicators with the worst outcomes were: identifies medication that interferes with urinary control; empties bladder completely; and urine leakage with increased abdominal pressure. The presence of nursing diagnosis MUI in the investigated women was related to a greater impairment of NOC indicators voids in appropriate receptacle, gets to toilet between urge and passage of urine, and empties bladder completely. Thus, urinary impairment was worse in women with elements of stress and urge urinary incontinence.
